# Melatonin Is a Promising Silage Additive: Evidence From Microbiota and Metabolites

**DOI:** 10.3389/fmicb.2021.670764

**Published:** 2021-05-26

**Authors:** Mao Li, Renlong Lv, Lidong Zhang, Xuejuan Zi, Hanlin Zhou, Jun Tang

**Affiliations:** ^1^Tropical Crops Genetic Resources Institute, Chinese Academy of Tropical Agricultural Sciences, Danzhou, China; ^2^Key Laboratory of Ministry of Education for Genetics and Germplasm Innovation of Tropical Special Trees and Ornamental Plants, Key Laboratory of Germplasm Resources of Tropical Special Ornamental Plants of Hainan Province, College of Forestry, Hainan University, Danzhou, China

**Keywords:** melatonin, stylo silage, metabolomics, bacterial community, fermentation quality

## Abstract

The safe and effective storage of forage are very important. As an important storage method, ensiling can keep fresh forage for a long time with less nutritional loss. Melatonin has antioxidant and bacteriostasis, usually used as a natural preservative. The influence of melatonin on silage microbial or fermentation quality has not been clarified. In the present study, we aimed to clarify whether melatonin affected stylo (*Stylosanthes guianensis*) silage quality via microbiota and metabolites. Melatonin addition significantly improved the silage fermentation quality, including the increased contents of lactic acid and total acid (244.18–255.81% and 63.95–78.97%, respectively), as well as the decreased in pH and butyric acid content compare with control group. Moreover, 16S rRNA sequencing indicated that melatonin addition enhanced the silage microbial diversity indices (such as increase in Shannon indices but decrease in Simpson indices), and significantly shaped the composition of silage microbiota (such as increased abundances of *Pantoea*, *Stenotrophomonas*, *Sphingobacterium*, and *Pseudomonas*, and decreased abundance of *Weissella*). Melatonin addition also dramatically affected the metabolites of sylo silage, such as raised malonic acid and some amino acid metabolism(glycine, threonine, methionine and ornithine), while reduced nucleic acid metabolism(2-deoxyuridine and thymine) and carbon metabolism(allose and 2-deoxy-D-glucose). Collectively, our results confirmed that the lowest melatonin addition (5 mg/kg) could improve the fermentation quality, and the potential mechanisms might be associated with the microbiota and metabolites in stylo.

## Introduction

As a naturally occurring indoleamine, melatonin is widely distributed in animals and plants, and it has a critical function in regulating their growth and development (Reiter et al., 2015). Melatonin has been studied deeply in animals, which is reported to be involved in a variety of physiological regulation processes, such as circadian rhythm, photoperiodic response, anti-aging response, immune function and oxidative stress (Tan et al., 2003, 2012). It is often used to improve sleep quality and employed as antioxidant to treat neurasthenia (Reiter et al., 2015, 2016). Recently, the biological function of melatonin in plants has also attracted wide attention. Melatonin is considered as an active oxygen scavenger, which can effectively scavenge reactive oxygen radicals, inhibit peroxidation, delay plant senescence, and alleviate salt, drought, heavy metal, cold, pathogens and other stresses (Kang et al., 2010; Yin et al., 2013; Zhang et al., 2013; Bajwa et al., 2014; Lee et al., 2014; Wang et al., 2014; Wei et al., 2015, 2017, 2020).

In recent years, more and more attention has been paid to food safety. Therefore, how to store crop products safely and effectively has become a hot research topic. Melatonin, as a natural preservative, has freshness-retaining effect, and it is widely used in the storage of fruits, vegetables and other crops. Melatonin can promote the ripening and quality of tomato fruit (Sun et al., 2014), delay the postharvest senescence, improve the cold resistance of peach fruit (Cao et al., 2016; Gao et al., 2017), reduce the postharvest decay of strawberry fruit and retain its nutritional value (Aghdam et al., 2019), and reduce the physiological degradation of cassava root after harvest (Ma et al., 2016).

Animal products are an important source of food, and feed safety is closely related to the quality of animal products. Under the background of global climate change, how to keep green forage safely and efficiently has become a great challenge for animal husbandry all over the world. Ensiling is an approach for long-term preservation of green forage under the anaerobic conditions, which is an ancient agricultural production approach used more than 3,000 years (Wilkinson et al., 2003; Kung et al., 2018). Generally, the characteristics of desirable silage were lower pH value and higher lactic acid content. Because both pH and organic acids (especially lactic acid) were important indexes to evaluate silage fermentation quality. In particular, the desirable legumes forage silage(Dry matter was <30–35%), the pH should be below 4.3–4.5, and the organic acids were mainly lactic acid (6–8%), that means forage well preservation(Kung et al., 2018).

Additives have been widely used to enhance the preservation of silage. Common additives include lactic acid bacteria (LAB) or other inoculants, chemicals, and enzymes (Muck et al., 2018). Nevertheless, the activities of biological additives have higher requirements on the environment, and the chemical additives will cause certain environmental problems and animal health concerns (Muck et al., 2018). Melatonin, as a natural preservative, has reliable safety in fruit and food storage, and it also has great potential for silage additives (Sun et al., 2014; Cao et al., 2016; Ma et al., 2016; Gao et al., 2017; Aghdam et al., 2019). However, the effect and mechanism of melatonin on silage fermentation quality are still unclear.

Stylo (*Stylosanthes guianensis*) is a very important legume forage widely distributed in the tropical and subtropical regions of the world, which is an important feed source for local livestock (Li et al., 2017). Due to the influence of climate and environmental factors, stylo production is highly seasonal, which limits its popularization and utilization and ensiling is really essential to ensure the balanced annual supply of livestock feed. Conventional additives have been reported in stylo silage, which can improve the fermentation quality and affect the microbial community of silage (Li et al., 2017; He et al., 2020c). In view of the characteristics of melatonin and its application in fruit and food preservation, we hypothesized that melatonin could regulate the fermentation quality of stylo silage by altering the microorganisms and metabolites. However, the effect of melatonin on silage fermentation of stylo is unknown. Herein, in this study, we detected the fermentation quality, microbial community and metabolites of stylo silage treated with different level of melatonin, in order to explore the effect of melatonin on silage and its possible mechanism.

## Materials and Methods

### Silage Processing

Stylo was cultivated at our experimental base (109°58’E, 19°52’N, Chinese Academy of Tropical Agricultural Sciences). The vegetative period stylo was harvested and cut into tiny sections (about 2 cm). Melatonin (BC Grade, Purity ≥ 99.0%) was obtained from Sangon Biotech Co., Ltd. (Shangshai, China). Four different treatments were conducted in our current study as follows: (Reiter et al., 2015) no additive (CK), (Tan et al., 2003) 5 mg/kg melatonin (Mela1) (Tan et al., 2012), 10 mg/kg melatonin (Mela2), and (Reiter et al., 2016) 20 mg/kg melatonin (Mela3). The application rate of melatonin was calculated based on fresh matter. Every treatment was carried out in triplicate. Briefly, 500 g of stylo and melatonin (powder) was blended and shook well, and the mixture was placed and vacuumed in plastic bags (50 cm × 20 cm × 10 cm; Guozhong Packing Co., Ltd., Haikou, China). A total of 12 bags (four treatments × three replicates) were prepared and incubated at normal temperature (25–30°C). The organic acid, microbial community and metabolites were determined after 30 days of fermentation.

### Chemical Analysis

Specimens were heated at 65°C for 72 h, and dried materials were ground for chemical analysis. The contents of dry matter (DM), crude protein (CP), water-soluble carbohydrates (WSC), neutral detergent fiber (NDF) and acid detergent fiber (ADF) were examined using previously established methods (He et al., 2020c). The contents of DM, CP, WSC, NDF and ADF in stylo before ensiled were 30.10, 10.49, 1.23, 51.04, and 40.76%, respectively. The fermentation quality of silage was determined using distilled water extracts. Briefly, 50 g wet silage was blended with 200 mL distilled water, followed by incubation at 4°C for 24 h and then filtration for analyzing. The pH and contents of lactic acid, acetic acid, propionic acid, butyric acid and ammonia-N were determined using previously established methods (Li et al., 2019).

### Microbial Diversity Analysis

#### DNA Isolation and 16S rRNA Gene Sequencing

The above-mentioned extracts were used for the molecular analysis of the microbiota. Microbial DNA was isolated from silage specimens with the E.Z.N.A.^®^ soil DNA Kit (Omega Bio-Tek, Norcross, GA, United States) according to manufacturer’s instructions. The concentration and purity of extracted DNA were assessed by NanoDrop 2000 UV-vis spectrophotometer (Thermo Scientific, Wilmington, United States), and DNA integrity was confirmed by electrophoresis on 1% agarose gel. Primers 338F (5’-ACTCCTACGGGAGGCAGCAG-3’) and 806R (5’-GGACTACHVGGGTWTCTAAT-3’) were adopted to amplify the V3–V4 hypervariable regions of the bacterial 16S rRNA gene using thermocycler PCR system (GeneAmp 9700, ABI, United States). After PCR products were purified and quantified, next-generation sequencing was carried out using Illumina MiSeq 2500 platform (Illumina, Inc., San Diego, CA, United States), and paired-end reads of 250 bp were generated.

#### Processing and Analysis of Sequencing Data

The assembly of tags was carried out using filtered reads according to the principles as follows: overlap between paired-end reads should be more than 10-bp overlap and less than 2% mismatch. The unique tags were obtained by removing redundant tags using software MOTHUR (Schloss et al., 2009). The abundance was then determined using the resultant unique tags. The high-quality reads were grouped into operational taxonomic units (OTUs) defined at a similarity of 97%. Diversity metrics were determined using the core-diversity plug-in within QIIME2^1^ (Callahan et al., 2016). The microbial diversity within an individual sample was assessed using the alpha diversity indices, including observed OTUs, Chao1 richness estimator, Shannon diversity index, Simpson and ACE index. Beta diversity was analyzed to assess the structural variation of microbiota across specimens, and then principal component analysis (PCA) was determined (Vázquez-Baeza et al., 2013). The described methods were employed to identify the bacterial strains with different abundances among samples and groups (Segata et al., 2011). Unless specified above, parameters used in the analysis were set as default. The sequencing data were deposited in the Sequence Read Archive (SRA) under the accession number of PRJNA629094.

### Metabolite Analysis

#### Metabolite Extraction

Briefly, 100 μL sample was placed into a 1.5-mL tube, and 350 μL pre-cold methanol and 10 μL internal standard (L-2-chlorophenylalanine, 1 mg/mL stock) were added into the tube, followed by vortex mixing for 30 s. The mixture was subjected to ultrasonication for 10 min in ice water and then centrifuged at 12,000 *g* for 15 min at 4°C. Next, 100 μL supernatant was collected and placed into a new tube. To prepare the QC (quality control) sample, 70 μL of each sample was collected, pooled and evaporated in a vacuum concentrator. Subsequently, 40 μL of methoxyamine hydrochloride (20 mg/mL in pyridine) was added, followed by incubation at 80°C for 30 min, and then the mixture was derivatized by 50 μL of BSTFA regent (1% TMCS, v/v) at 70°C for 1.5 h. The sample was gradually cooled to room temperature, and 5 μL of FAMEs (in chloroform) was added to QC sample. All specimens were then subjected to gas chromatograph coupled with a time-of-flight mass spectrometer (GC-TOF-MS).

#### GC-TOF-MS Analysis

An Agilent 7,890 gas chromatograph coupled with a time-of-flight mass spectrometer was adopted for GC-TOF-MS analysis, and a DB-5MS capillary column was employed in such system. Briefly, 1 μL aliquot of specimen was injected in splitless mode. Helium was employed as the carrier gas, the front inlet purge flow was set at 3 mL min^–1^, and the gas flow rate through the column was set at 1 mL min^–1^. The initial temperature was maintained at 50°C for 1 min, and then it was raised to 310°C at a rate of 10°C min^–1^ and kept at 310°C for 8 min. The temperatures of injection, transfer line, and ion source were set at 280, 280, and 250°C, respectively. The energy was −70 eV in electron impact mode. The mass spectrometry data were acquired in full-scan mode within the m/z range of 50–500 at a rate of 12.5 spectra per second after a solvent delay of 6.25 min.

#### Data Preprocessing and Annotation

Raw data analysis, including peak extraction, baseline adjustment, deconvolution, alignment and integration, was completed with Chroma TOF (V 4.3x, LECO) software, and LECO-Fiehn Rtx5 database was adopted to identify metabolites by matching the mass spectrum and retention index. Finally, the peaks found in less than half of QC samples or RSD > 30% in QC samples were eliminated.

#### Metabolite Data Analysis

The metabolite data were analyzed according to previous work (Ren et al., 2018; Xu et al., 2019). All specimens were tested using PCA and PLS-DA models. Differentially expressed metabolites (DEMs) (*P* < 0.05) were identified using the OPLS-DA model with first principal-component of VIP (variable importance in the projection) values (VIP > 1) in combination with Student’s *t*-test. Pearson correlation coefficients were calculated to examine the correlation between metabolite and relative abundance of microbial groups. The heatmap was generated by the corrplot package in R and performed using BMKCloud^2^.

### Statistics

The impacts of application rate were investigated using one-way analysis of variance in SAS 9.3 software (SAS Institute Inc., Cary, NC, United States). Duncan’s multiple range test was adopted to identify significant differences, and *P* < 0.05 was considered statistically significant.

## Results

### Fermentation Quality of Stylo Silage Treated With Melatonin

Table 1 illustrates the fermentation characteristics of stylo silage after fermentation. The pH of melatonin-treated silages was reduced compared with the CK group, and the lowest (*P* < 0.05) pH value was observed in the Mela1 group. Melatonin treatments remarkably (*P* < 0.05) elevated lactic acid contents, and the lowest lactic acid level was found in the CK group (*P* < 0.05), while there was no obvious difference among melatonin-treated groups. The acetic acid contents were no significant difference was detected (*P* > 0.05). Melatonin treatments dramatically (*P* < 0.05) reduced the levels of propionic acid and butyric acid, and their highest levels were both detected in the CK group (*P* < 0.05). Furthermore, no butyric acid was detected in three melatonin-treated groups. Melatonin treatments remarkably (*P* < 0.05) elevated total acid contents, and the lowest level was detected in the CK group (*P* < 0.05), while three melatonin-treated groups had no significant difference (*P* > 0.05). In addition, melatonin dramatically (*P* < 0.05) reduced the levels of NH_3_-N, and the highest levels was detected in the CK group (*P* < 0.05). The above-mentioned results revealed that the melatonin treatments could promote the fermentation quality, and there was no dramatic difference among melatonin-treated silages.

**TABLE 1:** Fermentation quality of Stylo silage supplemented with melatonin.

	**pH**	**Lactic acid (% DM)**	**Acetic acid (% DM)**	**Propionic acid (% DM)**	**Butyric acid (% DM)**	**Total acid (% DM)**	**NH_3_-N (% DM)**
CK	4.53 ± 0.04^*a*^	1.72 ± 0.13^*b*^	3.70 ± 0.27^*a*^	0.16 ± 0.03^*a*^	0.08 ± 0.03^*a*^	5.66 ± 0.25^*b*^	5.27±^*a*^
Mela1	4.20 ± 0.05^*b*^	5.92 ± 0.24^*a*^	3.32 ± 0.37^*a*^	0.06 ± 0.06^*b*^	0.00	9.30 ± 0.42^*a*^	4.11±^*b*^
Mela2	4.21 ± 0.02^*b*^	6.04 ± 0.13^*a*^	4.05 ± 0.44^*a*^	0.04 ± 0.01^*b*^	0.00	10.13 ± 0.44^*a*^	4.08±^*b*^
Mela3	4.27 ± 0.04^*b*^	6.12 ± 0.29^*a*^	3.10 ± 0.22^*a*^	0.06 ± 0.01^*b*^	0.00	9.28 ± 0.17^*a*^	4.09±^*b*^

*CK, control; Mela1, 5 mg/kg Melatonin; Mela2, 10 mg/kg Melatonin; Mela3, 20 mg/kg Melatonin. Means within the same column with different letters are significantly different (P < 0.05).*

### Microbiota Community of Stylo Silage Treated With Melatonin

A total of 913,107 raw reads and 883,046 raw tags were generated. After sequencing data were processed, averagely 73, 489 clean tags and 64,108 effective tags were obtained in each silage sample.

**FIGURE 1:**
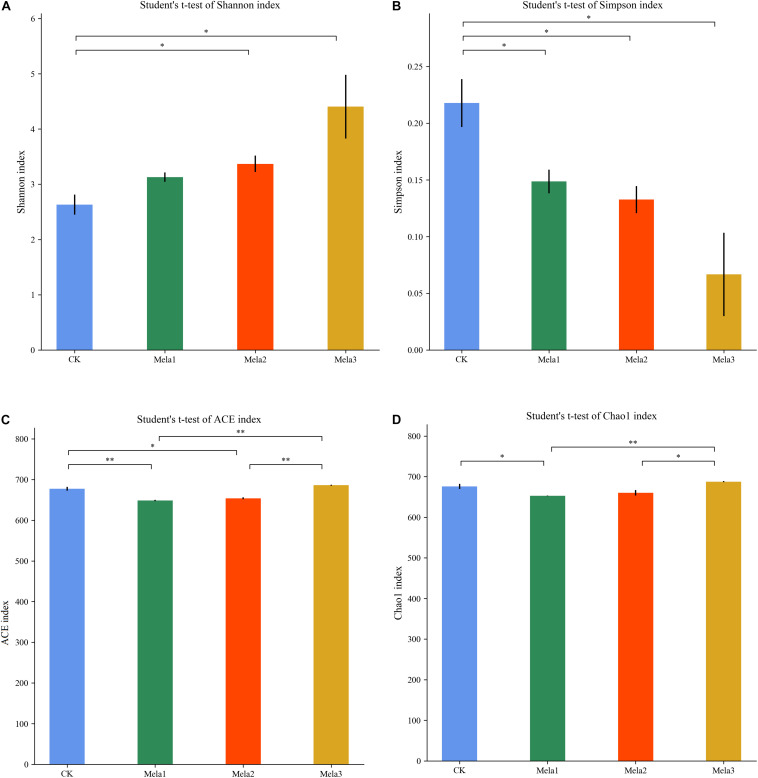
Alpha-diversity of bacterial diversity of stylo silage supplemented with melatonin (*n* = 3).

**FIGURE 2:**
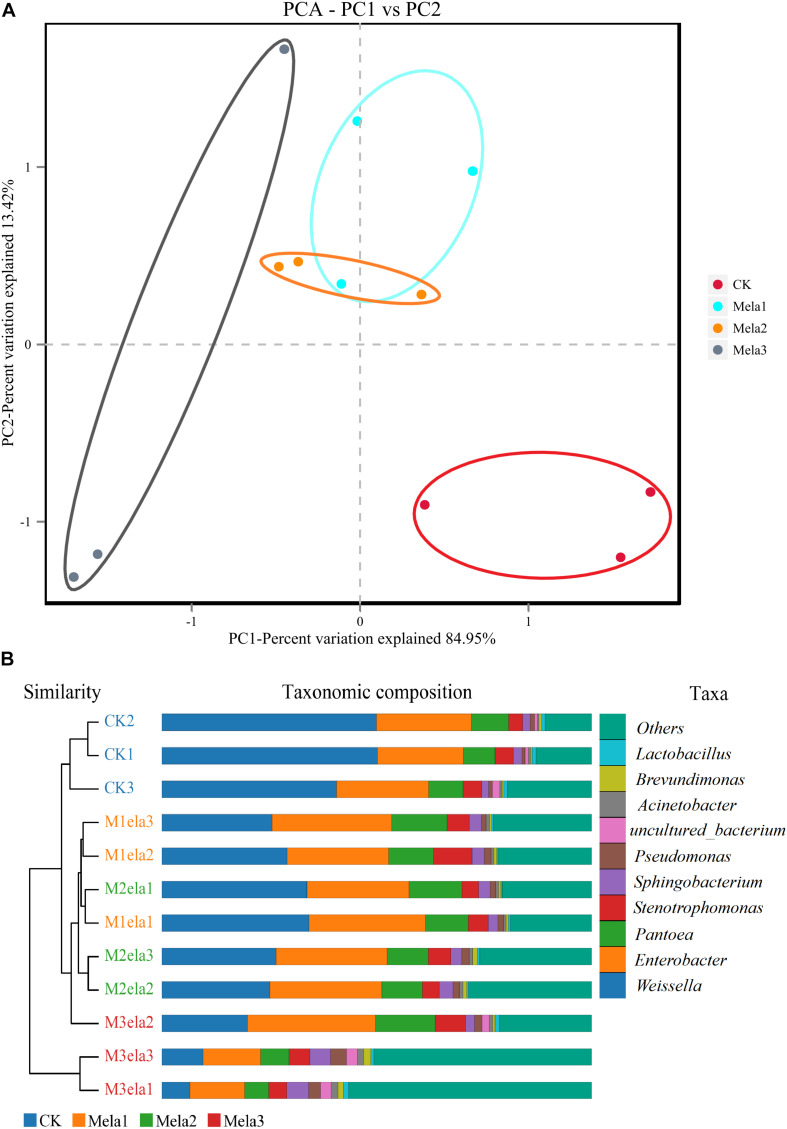
Beta-diversity of bacterial diversity of stylo silage supplemented with melatonin (*n* = 3).

Figure 1 shows the α-diversity of the bacterial community of silages. Melatonin treatment affected the Ace, Chao 1, Shannon and Simpson indices of microbial diversity and richness (Figure 1). The richness indices (Chao 1 and ACE) were not significantly different between the CK and Mela3 groups, while they were significantly higher compared with the Mela1 and Mela2 groups (*P* < 0.05). These indices showed that melatonin decreased the richness. In contrast to the richness indices, however, the Shannon index was higher, and the Simpson index in the additive-treated groups was lower than that in the CK group (*P* < 0.05), suggesting that melatonin treatments resulted in a higher diversity. Generally speaking, the high OTU number, Shannon index, as well as the low Simpson index indicate a higher microbial abundance and a higher diversity. The PCA and cluster tree were employed to examine the correlations among the community structures of the silage microbial community. A clear separation and difference of bacterial communities were found in four groups (Figure 2A), the CK and Mela3 groups had further genetic distance compared with the Mela1 and Mela2 groups (Figure 2B), and these results suggested that the microbial composition was changed in the silage process due to melatonin supplementation. Therefore, we drew a conclusion based on the α-diversity and β-diversity that the melatonin supplementation could affect the microbial diversity and community structure of stylo silage.

**FIGURE 3:**
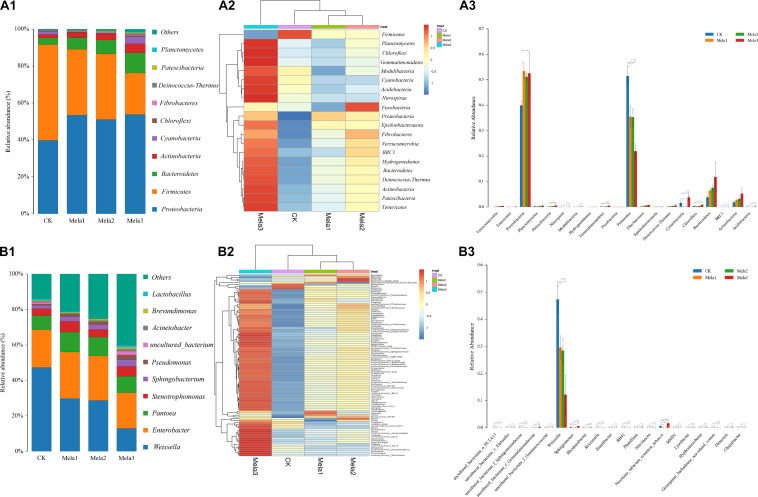
The bacterial abundance at the phylum and genus levels in stylo silage supplemented with melatonin (*n* = 3).

**FIGURE 4:**
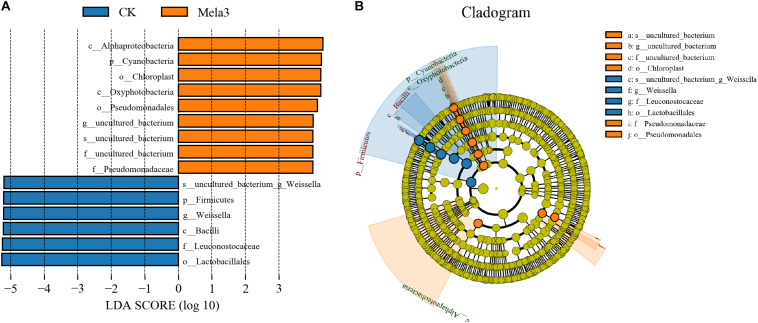
Comparison of microbial variations using the LEfSe online tool for stylo silage supplemented with melatonin (*n* = 3).

Figure 3A1,2 describes the microbial community at the phylum level. *Proteobacteria*, *Firmicutes*, *Bacteroidetes*, and *Actinobacteria* were the dominant phyla in all groups. The community was shifted along with the melatonin treatments (Figure 3A3), the abundances of *Proteobacteria*, *Bacteroidetes*, and *Actinobacteria* were increased, while the abundance of *Firmicutes* was decreased in additive-treated groups compared with the CK group (*P* < 0.05). To further investigate the effects of additives on microbial community during ensiling, we examined the bacterial structures of stylo silage at the genus level (Figure 3B1,2). *Weissella*, *Enterobacter*, *Pantoea*, *Stenotrophomonas*, and *Sphingobacterium* were the predominant genera in the four groups. The abundance of *Weissella* was decreased along with the ensiling process (Figure 3B3), while the abundances of *Enterobacter*, *Pantoea*, *Stenotrophomonas* and *Sphingobacterium*, *Pseudomonas*, *Acinetobacter*, *Brevundimonas*, and *Lactobacillus* were increased (except for *Enterobacter* in the Mela3 group). In addition, the number of unclassified species was obviously increased from 14.09% (CK) to 40.17% (Mela3). The differences in microbial community among groups were detected using the linear discriminant analysis (LDA) effect size (LEfSe) method, and the specific bacterial flora in each group was explored (LDA score > 4.0). Figures 4A,B shows that melatonin exerted a dramatic impact on the microbial community. Melatonin supplementation significantly elevated the relative abundance of *Pseudomonadaceae*, and reduced the abundance of *Weissella*. *Weissella* was the most abundant genus in the CK group, and *Pseudomonadaceae* was most abundant family in the Mela3 group, which could be identified as the specific bacterial taxa associated with melatonin.

Moreover, we showed that melatonin shaped the silage microbiota, including an increase in Shannon index but a decrease in Simpson index, implying that melatonin treatment could raise the microbial diversity. Meanwhile, the β-diversity analysis found distinct differences in microbia composition between the CK and melatonin-treated groups, and such differences were further amplified when the amount of added melatonin was increased. In addition, melatonin changed the microbial composition of stylo silage, the abundance of *Weissella* was decreased, and the abundances of unclassified species were increased along with the melatonin treatment.

### Metabolism of Stylo Silages Treated With Melatonin

**FIGURE 5:**
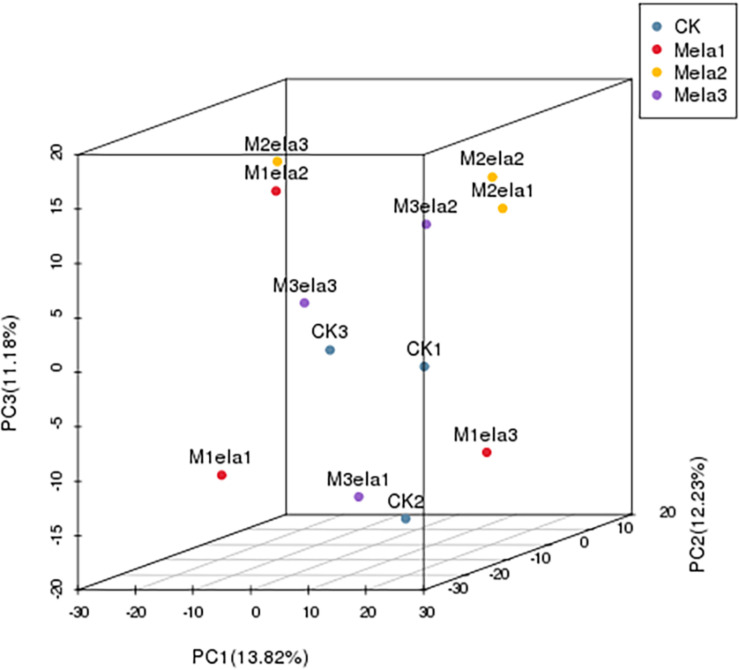
PCA (3D) of metabolic profiles in stylo silage supplemented with melatonin (*n* = 3).

**FIGURE 6:**
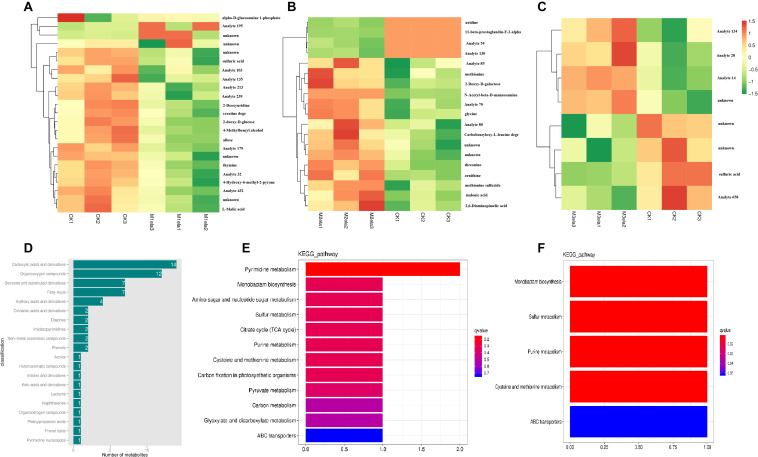
Melatonin affects the metabolism of stylo silage supplemented with melatonin (*n* = 3). Heatmap analysis of different metabolites identified from GC-MS analysis in stylo silage after melatonin addition (**A**, CK vs. Mela1; **B**, CK vs. Mela2; **C**, CK vs. Mela3) (*n* = 3). **(D)** The top 20 metabolites were classified. KEGG analysis of different metabolites identified from GC-MS analysis in the silage after melatonin addition (**E**, CK vs. Mela1; **F**, CK vs. Mela3) (*n* = 3).

To clarify the impacts of melatonin addition on silage microbiota, we further determined the levels of metabolites in the silage specimens among four groups. According to PCA (3D), the metabolites in four groups of specimens were obviously segregated by PC1, PC2 and PC3, representing 13.82, 12.23, and 11.18% of variations among specimens with various additives, respectively (Figure 5). To analyze metabolites in the silage specimens, 214 metabolites were found from 500 peaks in the chromatograms, and 64 known metabolites were identified. These identified metabolites were divided into 21 categories, including carboxylic acids and derivatives, organooxygen compounds, benzene and substituted derivatives, fatty acyls, hydroxy acids and derivatives, cinnamic acids and derivatives, diazines, imidazopyrimidines, non-metal oxoanionic compounds, phenols, azoles, heteroaromatic compounds, indoles and derivatives, keto acids and derivatives, lactams, naphthalenes, organonitrogen compounds, phenylpropanoic acids, prenol lipids, pyrimidine nucleosides, and tropane alkaloids (Figure 6D).

Table 2 and Figures 6A–C list the identified potential markers. When the three melatonin-treated groups were compared with the CK group, the contents of 10 metabolites were significantly changed between the CK and Mela1 groups. For example, the contents of sulfuric acid (*P* = 0.019), 2-deoxyuridine (*P* = 0.049), 4-methylbenzyl alcohol (*P* = 0.04), thymine (*P* = 0.018), L-malic acid (*P* = 0.031), 4-hydroxy-6-methyl-2-pyrone (*P* = 0.041), allose (*P* = 0.038), creatine degr (*P* = 0.05), and 2-deoxy-D-glucose (*P* = 0.035) were down-regulated, while the content of alpha-D-glucosamine 1-phosphate (*P* = 0.018) was up-regulated. Moreover, the contents of nine metabolites were significantly altered between the CK and Mela2 groups. For example, the contents of malonic acid (*P* = 0.032), carbobenzyloxy-L-leucine degr (*P* = 0.036), glycine (*P* = 0.005), threonine (*P* = 0.001), methionine (*P* = 0.038), methionine sulfoxide (*P* = 0.008), 2-deoxy-D-galactose (*P* = 0.019), ornithine (*P* = 0.028), and 2,6-diaminopimelic acid (*P* = 0.034) were up-regulated. In addition, the content of one metabolite, sulfuric acid (*P* = 0.023), was significantly down-regulated between the CK and Mela3 groups. KEGG assay indicated that melatonin significantly changed the microbial functions, such as pyrimidine metabolism, monobactam biosynthesis, amino sugar and nucleotide sugar metabolism, sulfur metabolism, citrate cycle (TCA cycle), purine metabolism, cysteine and methionine metabolism, carbon fixation in photosynthetic organisms, pyruvate metabolism, carbon metabolism, glyoxylate and dicarboxylate metabolism and ABC transporters between the CK and Mela1 groups (Figure 6E). The monobactam biosynthesis, sulfur metabolism, purine metabolism, cysteine and methionine metabolism and ABC transporters were also altered between the CK and Mela3 groups (Figure 6F). Collectively, melatonin addition significantly altered the metabolism of silage microbiota.

**TABLE 2:** Candidate silage metabolites that were different between the CK and melatonin-treated groups.

**Compounds**	**Fold_change**	***P*-value**	**VIP**
**CK_vs._Mela1**			
Sulfuric acid	0.604	0.019	1.664
2-Deoxyuridine	0.709	0.049	1.557
4-Methylbenzyl alcohol	0.704	0.04	1.611
Thymine	0.692	0.018	1.687
L-Malic acid	0.726	0.031	1.58
4-Hydroxy-6-methyl-2-pyrone	0.729	0.041	1.633
Allose 1	0.658	0.038	1.568
Creatine degr	0.754	0.05	1.498
2-Deoxy-D-glucose 1	0.654	0.035	1.577
Alpha-D-glucosamine 1-phosphate	4.666	0.018	1.639
**CK_vs._Mela2**			
Malonic acid 1	1.296	0.032	1.998
Carbobenzyloxy-L-leucine degr1	1.29	0.036	1.984
Glycine 2	1.488	0.005	2.082
Threonine 1	1.611	0.001	2.197
Methionine 1	1.33	0.038	1.807
Methionine sulfoxide 2	1.488	0.008	2.075
2-Deoxy-D-galactose 2	1.293	0.019	1.991
Ornithine 1	1.666	0.028	2.072
2,6-Diaminopimelic acid 2	1.4	0.034	1.949
**CK_vs._Mela3**			
Sulfuric acid	0.715	0.023	2.116

*CK, control; Mela1, 5 mg/kg Melatonin; Mela2, 10 mg/kg Melatonin; Mela3, 20 mg/kg Melatonin. Fold change (fold change > 1 means melatonin < control; fold change < 1 means melatonin > control). N = 3, the data were analyzed by unpaired t-test. P-value, t-test significance. VIP, variable importance in the projection.*

### Correlations Between the Relative Abundances of Bacteria and Metabolites of Stylo Silages

**FIGURE 7:**
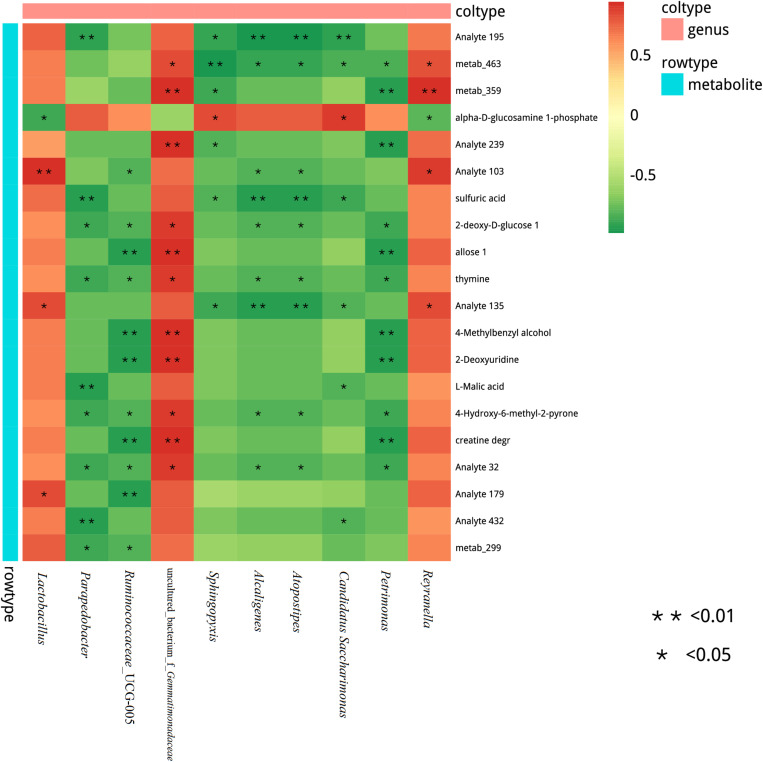
Correlation matrix between the silage differential metabolites affected by the melatonin and the differential microbiota at the genus level. Positive correlations are shown in red, and negative correlations are indicated in green. Color intensity is proportional to the correlation values (r). *P*-values are shown as *0.01 < *P* ≤ 0.05, **P ≤ 0.01.

We also assessed the correlation between the metabolites and microbiome of stylo silages. At the genus level, a Spearman correlation heatmap was created for the microbial community within the silages (Figure 7). Alpha-D-glucosamine-1-phosphate was positively associated with *Sphingopyxis* and *Candidatus Saccharimonas*, while it was negatively associated with *Lactobacillus*. Sulfuric acid was negatively associated with *Candidatus Saccharimonas*, *Atopostipes*, *Alcaligenes*, *Sphingopyxis*, and *Parapedobacter*. Moreover, 2-deoxy-D-glucose, thymine and 4-hydroxy-6-methyl-2-pyrone were positively correlated with uncultured_bacterium_f_*Gemmatimonadaceae*, while they were negatively correlated with *Petrimonas*, *Atopostipes*, *Alcaligenes*, *Ruminococcaceae*_UCG-005, and *Parapedobacter*. In addition, 4-methylbenzyl alcohol, 2-deoxyuridine and creatine degr were positively correlated with uncultured_bacterium_f_*Gemmatimonadaceae*, while they were negatively correlated with *Petrimonas* and *Ruminococcaceae*_UCG-005. L-Malic acid was negatively associated with *Candidatus, Saccharimonas*, and *Parapedobacter*. This study showed significant correlation between many different bacteria and the metabolites of the stylo silage. A great deal of metabolites were positively associated with LAB species, but negatively associated with undesirable bacteria during ensiling. Silage fermentation is a very complex biological process, which involves a large variety of microorganisms. For instance, the process produces many different metabolites, which can determine the fermentation quality.

## Discussion

### Melatonin Improves Fermentation Quality of Stylo Silage

Melatonin is widely used in postharvest storage of fruits, which can enhance the antioxidant activity and effectively retard the decline of fruit quality (Gao et al., 2016; Hu et al., 2017; Liu et al., 2018; Zhai et al., 2018; Zhang et al., 2018). In the present study, we showed that melatonin facilitated the silage quality of stylo through the following fermentation characteristics. The pH value of additive-treated silages was close to 4.2, ensuring the well preservation of stylo silage (Edwards and McDonald, 1978). A previous study has shown higher pH values at 5.0 or above in stylo silage, which is maybe caused melatonin treatment more efficient than *Moringa oleifera* leaves or gallic acid addition (He et al., 2020a, c). Lactic acid in silage is the dominant product from fermentation, which is an important index to evaluate silage quality. In the present study, melatonin promote the production of lactic acid, which increased 244.18–255.81% compare with control group, and the results were similar with other additives (Li et al., 2017; He et al., 2020a). In the current study, the level of butyric acid was not detectable in melatonin-treated silages, because melatonin has the effects of antioxidation and inhibition of undesirable bacteria, there were no *Clostridium* in the silage bacterial communities, indicating that the stylo was well preserved. Similar studies have been reported, (He et al., 2020a, b) reported that butyric acid was not detected in silage after adding gallic acid or tannic acid, thus improving the fermentation quality of silage. Gallic acid and tannic acid are polyphenolic antioxidants, which have antifungal and antiviral effects, and can inhibit the harmful microorganisms such as *Clostridium*, *Listeria*, and *Escherichia coli* in the process of silage (He et al., 2020a, b). The total acid content showed similar trend as lactic acid and acetic acid, while melatonin-treated silage improved total acid content 63.95–78.97% compared with the CK group after ensiling. In this study, melatonin treatment reduced the content of NH_3_-N of control stylo silage, the effect similar with gallic acid (He et al., 2020a). However, the NH_3_-N contents of stylo silage were various. One study reported the NH_3_-N in stylo and *Moringa oleifera* leaves mixed silage were range from 5.07 to 11.1%(He et al., 2020c). In contrary, gallic acid reduced NH_3_-N from 1.46 to 0.29% (He et al., 2020a). These differences may be caused by the different antioxidant or antibacterial ability of additives. These results indicated that melatonin addition promoted the fermentation quality, and the addition of various amounts of melatonin in this study showed similarly effect. Therefore, it is suggested that melatonin with a minimum dosage (5 mg/kg) can achieve the effect.

### Melatonin Addition Shapes Microbiota Community of Stylo Silage

In present study, the beta-diversity shown separation and difference of bacterial communities of stylo silage. These results suggested that the microbial composition was changed in the silage process due to melatonin treatments. The specific changes were reflected by the differences in bacterial composition at the genus level among different treatments stylo silage. Some previous reports consistent with this study, additives such as *Moringa oleifera powder* and gallic acid shaped the bacterial community structure (He et al., 2020a, c). In particular, the structure of the bacterial communities greatly at the genus level. As a lactic acid-producing bacterial strain, *Weissella* exists widely in fermented food or silage (Cai et al., 1998). Furthermore, it is the dominant group at the beginning of silage fermentation before it is replaced by other lactic acid-producing microorganisms after ensiling (Cai et al., 1998; Guan et al., 2018; Ding et al., 2020). This might be explained by the fact that the fermentation capacity of *Weissella* was weaker compared with other microorganisms, leading to weakened competitiveness and its replacement by *Pantoea*, *Stenotrophomonas*, *Sphingobacterium*, *Pseudomonas*, *Acinetobacter*, *Brevundimonas*, and *Lactobacillus*. *Enterobacter* is one of the major undesirable microorganisms in silage that produce acetic acid to raise the pH, which, as a result, reduces the fermentation quality (Ni et al., 2017; Wang et al., 2019; Lv et al., 2020). In this study, the abundance of *Enterobacter* was higher and similar in four groups, which was reflected by the relatively high level of acetic acid. Therefore, the effect of *Enterobacter* on stylo fermentation quality was slight. *Pantoea* (8–11.9%) was the third dominant strain in the present study, which was higher than *A. villosum* silage (1.44–4.56%) (Lv et al., 2020). Its effect on silage fermentation still has a dispute. Ogunade et al. (2018) have reported that *Pantoea* is negatively correlated with ammonia-N, and *Pantoea* is beneficial to silage fermentation. However, some studies have claimed that *Pantoea* is an undesirable microbial strain because it competes the fermentation substrate with LAB^22^. *Stenotrophomonas* and *Sphingobacterium* belong to aerobic or facultative anaerobic non-fermentative Gram-negative bacilli, which can utilize a variety of sugars and produce acid (Yabuuchi et al., 1983; Palleroni and Bradbury, 1993). *Pseudomonas* is considered as an undesirable bacterial strain for silage due to its possibility of biogenic amine production as well as decreased protein content and nutritional value (Santos Silla, 1996; Dunière et al., 2013). Ogunade et al. (2018) found that these four microorganisms were negatively correlated with pH, ammonium nitrogen, yeast and mold, suggesting that they may be beneficial to silage fermentation. Moreover, in this study, *Pantoea*, *Stenotrophomonas*, *Sphingobacterium, and Pseudomonas* have relative lower abundances, and which significantly increased in melatonin-treated groups. Therefore, the underlying mechanisms need to be further investigated. We speculated that the increased abundances of these microbial species enhanced the biodiversity of the silage micro-ecosystem, while increased the quantity and abundance of lactic acid producing microorganisms. These makes the silage micro-ecosystem more species-rich and conducive to the dynamic balance of beneficial and undesirable microorganisms, and then generated beneficial synergistic effect, leading to improved silage quality. Another possibility could be attributed to the increased number of unclassified microorganisms that affect the fermentation of silage. However, in-depth investigations are required to clearly understand their roles in silage fermentation. Therefore, the effects of melatonin treatment on microbial composition need to be further explored using Single Molecule, Real-Time (SMRT) Sequencing or metagenomic sequencing.

### Melatonin Addition Alters the Metabolism of Stylo Silages

The fermentation process of silage is complex, involving the interaction of many microorganisms mainly composed of lactic acid bacteria and their metabolites. Besides its effects on the composition of silage microbiota, melatonin also changed the metabolism of silage microbiota. For a long time, the studies on silage metabolism are few, and the type and quantity of metabolites in silage remain largely unclear. The currently available research mainly focuses on metabolites related to fermentation quality and aerobic stability, such as lactic acid, acetic acid, propionic acid, butyric acid and propanediol (Broberg et al., 2007). The impacts of additives on the silage metabolites are still unknown. Guo et al. (2018) have reported the metabolites in alfalfa silage, showing that the LAB treatments raise the desirable compounds of 2,3-butandiol, adenine and amino acids, but decrease the content of undesirable cadaverine, which can be attributed to the role of corruption, leading to bad fermentation quality. Xu et al. (2019) have found that there are more amino acids (phenylalanine, lysine, tyrosine and glycine), phenolic acids (4-hydroxycinnamic acid and 3,4-dihydroxycinnamic acid), flavoring agent (gluconic lactone) and organic acids (lauric acid, 3-hydroxypropionic acid, pentadecanoic acid, oxamic acid and isocitric acid) in whole crop corn silage treated with LAB. In the present study, melatonin supplementation up-regulated the contents of alpha-D-glucosamine-1-phosphate, malonic acid, carbobenzyloxy-L-leucine degr, glycine, threonine, methionine, methionine sulfoxide, 2-deoxy-D-galactose, ornithine and 2,6-diaminopimelic acid, while such supplementation down-regulated the contents of sulfuric acid, 2-deoxyuridine, 4-methylbenzyl alcohol, thymine, L-malic acid, 4-hydroxy-6-methyl-2-pyrone, allose, creatine degr and 2-deoxy-D-glucose. The increase of amino acid content was consistent with previous reports (Guo et al., 2018; Xu et al., 2019). Malonic acid is the intermediate for the production of vitamins and amino acids. These metabolites with beneficial effects could also be considered as an evaluation index for fermentation quality. The reduced metabolites, such as nucleic acid metabolites, and special sugars could be used as biomarkers of poor fermentation quality in stylo silage. The similar phenomenon has been reported in alfalfa silage that cadaverine suggests bad fermentation quality (Guo et al., 2018). Different metabolites affected various metabolic pathways, including pyrimidine metabolism, monobactam biosynthesis, amino sugar and nucleotide sugar metabolism, sulfur metabolism, citrate cycle, purine metabolism, cysteine and methionine metabolism, carbon fixation in photosynthetic organisms, pyruvate metabolism, carbon metabolism, glyoxylate and dicarboxylate metabolism and ABC transporters. In general, the addition of melatonin in stylo silage changed the composition of metabolites, increased the beneficial metabolites and promoted the silage fermentation quality. Nevertheless, it remains unclear whether the altered metabolites are merely the effect of melatonin on silage microbiota, or whether melatonin modulates the fermentation quality via these metabolites. Therefore, it is necessary to explore the mechanisms underlying the melatonin-induced changes in fermentation quality.

Spearman correlation analysis was carried out to explore the correlations between microbial and metabolites. Xu et al. (2019) have found that metabolites in corn silage are positively associated with LAB species but negatively associated with undesirable microorganisms. In this study, alpha-D-glucosamine-1-phosphate was phosphate derivatives of glucose, and the result reflected the ability of glucose utilization to some extent. Lactobacillus had a stronger ability than *Sphingopyxis* and *Candidatus Saccharimonas*, leading to the opposite correlation. Acidic environment will hinder the growth of pathogen and spoilage, which is negatively correlated with sulfuric acid. In addition, 2-deoxy-D-glucose and thymine can be used by bacteria through oxidation or fermentation. Therefore, they were negatively correlated. L-Malic acid was negatively associated with *Candidatus Saccharimonas* and *Parapedobacter*, because the lower pH could inhibit their growth. Generally, there were significant correlations between many different bacteria and the metabolites of the stylo silage, and it was mainly determined by the characteristics and functions of metabolites. Furthermore, fermentation process produces many different metabolites, which can determine the fermentation quality. It is worth noting that the metabolites with biological functions are negatively correlated with some lactic acid bacteria (species level) in silage, which may be due to the low concentration of metabolites in the sample, which cannot be accurately detected, but the low content of metabolites may still affect the silage quality and even the health of animals. Therefore, it is still of great significance to study the correlation between microorganisms and metabolites in stylo silage, which can be used to screen targeted additives to regulate silage fermentation and prepare high-quality silage. It is important to note that correlation is not causation, it is based on statistical data, the parameters are related, the results are only speculation. Nevertheless, it is of great significance to study the correlation between stylo silage microorganisms and metabolites with biological functions, which can provide a theoretical basis for screening targeted additives to regulate silage fermentation and prepare high-quality silage.

In summary, melatonin addition increased the quantity and abundance of lactic acid producing microorganisms, while raised the beneficial metabolites malonic acid and amino acid and reduced some nucleic acid and special sugars, and then affected the pathway of amino acid metabolism, nucleic acid metabolism and carbon metabolism etc. Finally, it inhibited undesirable microorganisms and raised the lactic acid content and dropped pH, improved the silage fermentation quality.

## Conclusion

Melatonin addition significantly improved silage quality of stylo, and the lowest addition (5 mg/kg) showed the beneficial effect, including the increased contents of lactic acid and total acid, as well as the decrease in pH and butyric acid. Additionally, besides its effects on enhancing the micrbial diversity (such as the increase in shannon indices but the decrease in simpson indices), melatonin significantly shaped the composition of silage microbiota (such as the increased abundances of *Pantoea*, *Stenotrophomonas*, *Sphingobacterium*, and *Pseudomonas*, and the decreased abundance of *Weissella*), and the metabolism of silage microbiota. Moreover, Melatonin addition also dramatically affected the metabolites of sylo silage, such as raised malonic acid and some amino acid metabolism(glycine, threonine, methionine and ornithine), while reduced nucleic acid metabolism(2-deoxyuridine and thymine) and carbon metabolism(allose and 2-deoxy-D-glucose). Therefore, the analysis of microbiome and metabonomics of stylo silage can improve our understanding of the biological process of silage fermentation, and it is also conducive to scientifically evaluate and regulate the silage quality.

## Data Availability Statement

The datasets presented in this study can be found in online repositories. The names of the repository/repositories and accession number(s) can be found below: The sequencing data were deposited in the Sequence Read Archive (SRA) under the accession number of PRJNA629094.

## Author Contributions

ML, XZ, and JT did the experimental design work. ML, XZ, RL, LZ, and JT conducted the experiments. ML, XZ, RL, LZ, JT, and HZ analyzed the data. ML and XZ wrote the manuscript. All authors read and approved the manuscript.

## Conflict of Interest

The authors declare that the research was conducted in the absence of any commercial or financial relationships that could be construed as a potential conflict of interest.

## Footnotes

1 https://docs.qiime2.org/2019.1/

2 http://www.biocloud.net/
